# Can EEG and MEG detect signals from the human cerebellum?

**DOI:** 10.1016/j.neuroimage.2020.116817

**Published:** 2020-07-15

**Authors:** Lau M. Andersen, Karim Jerbi, Sarang S. Dalal

**Affiliations:** a Center of Functionally Integrative Neuroscience, Aarhus University, Denmark; b NatMEG, Karolinska Institutet, Stockholm, Sweden; c Computational and Cognitive Neuroscience Lab (CoCo Lab), Psychology Department, University of Montreal, Montreal, QC, Canada; d MEG Unit, University of Montreal, Montreal, QC, Canada

## Abstract

The cerebellum plays a key role in the regulation of motor learning, coordination and timing, and has been implicated in sensory and cognitive processes as well. However, our current knowledge of its electrophysiological mechanisms comes primarily from direct recordings in animals, as investigations into cerebellar function in humans have instead predominantly relied on lesion, haemodynamic and metabolic imaging studies. While the latter provide fundamental insights into the contribution of the cerebellum to various cerebellar-cortical pathways mediating behaviour, they remain limited in terms of temporal and spectral resolution. In principle, this shortcoming could be overcome by monitoring the cerebellum’s electrophysiological signals. Non-invasive assessment of cerebellar electrophysiology in humans, however, is hampered by the limited spatial resolution of electroencephalography (EEG) and magnetoencephalography (MEG) in subcortical structures, i.e., deep sources. Furthermore, it has been argued that the anatomical configuration of the cerebellum leads to signal cancellation in MEG and EEG. Yet, claims that MEG and EEG are unable to detect cerebellar activity have been challenged by an increasing number of studies over the last decade. Here we address this controversy and survey reports in which electrophysiological signals were successfully recorded from the human cerebellum. We argue that the detection of cerebellum activity non-invasively with MEG and EEG is indeed possible and can be enhanced with appropriate methods, in particular using connectivity analysis in source space. We provide illustrative examples of cerebellar activity detected with MEG and EEG. Furthermore, we propose practical guidelines to optimize the detection of cerebellar activity with MEG and EEG. Finally, we discuss MEG and EEG signal contamination that may lead to localizing spurious sources in the cerebellum and suggest ways of handling such artefacts.

This review is to be read as a perspective review that highlights that it is indeed possible to measure cerebellum with MEG and EEG and encourages MEG and EEG researchers to do so. Its added value beyond highlighting and encouraging is that it offers useful advice for researchers aspiring to investigate the cerebellum with MEG and EEG.

## Highlights

• Electro- and magnetoencephalography can detect cerebellar signals.
• We propose guidelines for detecting cerebellar signals non-invasively.
• The cerebellum subserves several functions beyond mere motor processing.

## 1 Introduction

In addition to its well-established role in the control and coordination of motor behaviour, the cerebellum is involved in sensory processing (audition: Petacchi et al., 2005; retinotopy: van Es et al., 2019) and cognitive tasks ranging from learning and memory to higher order cognitive control processes (Ito, 1984; Thaut, 2003; Bellebaum and Daum, 2007; Strick et al., 2009; Casabona et al., 2010; Stoodley et al., 2012; Buckner, 2013). King et al. (2019), using functional magnetic resonance imaging (fMRI), recently showed that the cerebellum is involved in functions as diverse as hand movements, saccades, divided attention, verbal fluency, autobiographical recall, word comprehension, action observation, mental arithmetic, emotion processing and language processing, among other functions. This is further evidence, if any were needed, that we simply cannot afford to ignore the cerebellum in studies of human brain processes. However, the utility of noninvasive electrophysiological techniques like electroencephalography (EEG) and magnetoencephalography (MEG) for measuring cerebellar responses has not been clearly established, and sometimes even explicitly discounted in textbooks (Tyner et al., 1989; Covey and Carter, 2015). Meanwhile, studies employing EEG or MEG to delineate brain networks often do not consider the cerebellum as a potential source of the measured responses. In this review, we argue for a more optimistic view on EEG’s and MEG’s ability to detect cerebellar activity. We furthermore offer advice for how to improve cerebellar recordings with MEG, hopefully providing a valuable tool that other researchers aspiring to record the electrophysiological signals of the cerebellum can rely on.

Our current knowledge of the electrophysiological mechanisms that mediate cerebellar activity comes mainly from direct recordings in animals. Investigations of the human cerebellum consist predominantly of studies in patients with cerebellar lesions or studies tracking metabolic or haemodynamic processes such as positron emission tomography (PET) and fMRI. In contrast to electrophysiological recordings, these neuroimaging techniques only provide an indirect measure of neural activity by monitoring local metabolic or haemodynamic responses. This notwithstanding, neuroimaging studies using these modalities play a pivotal role in elucidating the functional role of the cerebellum by unravelling its contribution to numerous tasks such as motor control, visually guided behaviour and many cognitive tasks (Buckner, 2013). Because they monitor the activity of the whole brain simultaneously, these imaging techniques are also used to examine the involvement of the cerebellum in potential large-scale cerebral networks and to assess the functional-role of cerebellar-thalamo-cortical pathways (Diedrichsen et al., 2019).

Nevertheless, the relatively sluggish nature of haemodynamic and metabolic responses remains a severe limitation when it comes to investigating the precise temporal properties of cerebellar activity. Recording signals from the cerebellum with temporal resolution comparable to that obtained in electrophysiology (i.e., millisecond range) is crucial in order to correlate the measured activity with behavioural parameters (such as reaction times or time-varying movement parameters) but also in order to compare activation latencies between cerebellum and other brain structures and finally to assess putative fine-grained synchronization properties between the cerebellum and various nodes of the involved cerebral network. To achieve the above, one would require a non-invasive technique that provides millisecond temporal resolution combined with whole-head coverage. EEG and MEG fulfil these requirements. While the former measures the electrical potentials on the scalp, the latter detects the minute magnetic signals generated on the surface by the same underlying cerebral generators (Hämäläinen et al., 1993). Both EEG and MEG record brain signals with millisecond resolution and currently available systems provide dense channel arrays with up to approximately 300 recording sites yielding full coverage of the head.

But do these methods provide the optimal spatiotemporal resolution at which to study the physiology of the human cerebellum? Unfortunately, the answer to this question is not straightforward. One problem lies with the poor spatial resolution of these techniques in deep structures, i.e., structures located far from the sensors. The distance from the sensor array and signal diffusion issues yield a low signal-to-noise ratio (SNR) and linear mixing at the individual recording sites. As a result, from a source estimation perspective, superficial sources (e.g., sources in primary auditory or somatosensory cortices) are easier to localize non-invasively with MEG or EEG than sources located in deeper brain structures (e.g., hippocampus or deeper substructures of the cerebellum). Furthermore, it has been speculated that the neuronal architecture of the cerebellar cortex may also be a specific limiting factor preventing detection of cerebellar sources with non-invasive methods due to signal cancellation. In general, MEG and EEG signals arise from (a) the spatial summation of underlying neural activity, i.e. many postsynaptic potentials in neighbouring dendrites with similar orientations, and (b) the temporal summation of these potentials, i.e. that these currents arise in the same short time interval. For the cerebellum, it has been thought that due to its more intricate folding relative to cerebral cortex, signals would cancel one another because neighbouring patches of cerebellum activation would result in currents of opposing orientation. This cancellation would preclude the spatial summation needed to generate a signal detectable with EEG or MEG. These potential difficulties, together with the attenuation of MEG and EEG signal strength with depth, has led to the prevailing view that MEG and EEG are not suitable for the detection of cerebellar activity. As a result, sources that appear to be localized in cerebellum are often suspected of being artefactual in origin or simply resulting from noisy data. Nonetheless, a small but increasing number of MEG and EEG studies report activations in the cerebellum in a range of tasks. So can MEG and EEG detect cerebellar activity after all? And if so, how can we optimize its detection and how can we rule out false positives? We believe that there is now sufficient evidence in the literature to address these questions.

## 2 Why is the detection of cerebellar activity with EEG and MEG a controversial issue?

**Fig. 1:**
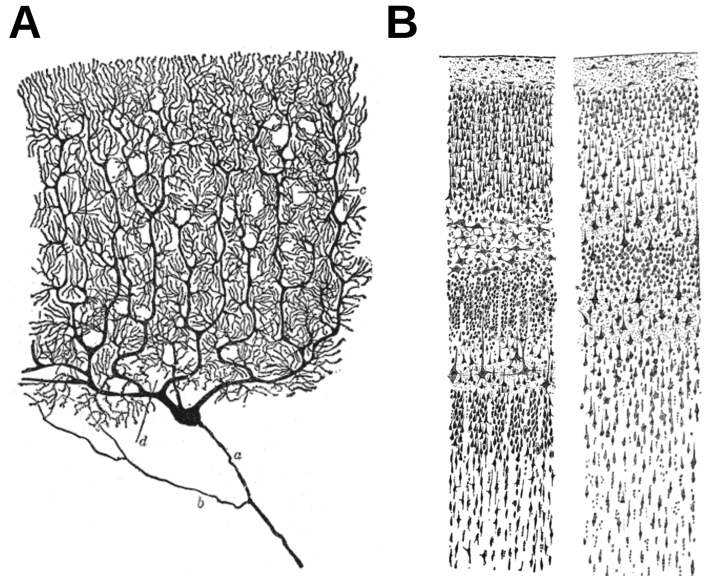
**Similarities between Purkinje cells (cerebellum) and pyramidal cells (cerebral cortex) A)** a sketch of a Purkinje cell from the human cerebellum. **B)** a sketch of the pyramidal cells in sensory cortex and motor cortex of an adult, showcasing the different cortical layers. Both sketches are by Ramon y Cajal and are public domain:https://en.wikipedia.org/wiki/File:Purkinje_cell_by_Cajal.png andhttps://commons.wikimedia.org/wiki/File:Cajal_cortex_drawings.png.

It has been suggested that it has been difficult to record cerebellar activity with noninvasive EEG (and by extension, MEG) since the neurons of the cerebellum are arranged in a “closed field” configuration (Bantli, 1972). However, the arrangement of Purkinje cells in cerebellar cortex (Ramón y Cajal, 1904) is very analogous to that of pyramidal cells in cerebral cortex (see Fig. 1) and likely contribute to the scalp EEG/MEG signal. Studies on the turtle cerebellum have demonstrated that an external magnetic field can be detected at a distance; a field of 1 ​pT was detected at a distance of 17 ​mm when a cerebellar patch of 10 ​mm^3^ was activated (Okada et al., 1987). The structure of the turtle cerebellar cortex is very similar to that of higher species, including humans (Eccles, 2013). Buzsáki et al. (2012) furthermore highlight that the cerebellum has an ordered structure, which would result in an open field configuration, but they note that cerebellar activation is mainly local, meaning that corresponding external magnetic fields are weak. However, in cases where synchronous activity is imposed on the cerebellum from outside itself, magnetic fields strong enough to be detected by MEG can be generated, as for example is the case in epilepsy (Kandel and Buzsáki, 1993). As synchronous activity may also be imposed on the cerebellum by direct brain stimulation methods routinely used in neuroscience, it should, at least in principle, not be impossible to detect cerebellum with MEG or EEG. MEG may be a more appropriate modality than EEG, however, because MEG source localization suffers less from inaccuracies of the head model than EEG source localization does. This makes source localization with MEG more precise and accurate than with EEG given the same quality of head model (Hämäläinen et al., 1993; Vorwerk et al., 2014; Wolters et al., 2006). Also, specifically for high-frequency oscillations, MEG appears to capture them with higher fidelity than EEG does (Muthukumaraswamy and Singh, 2013).

One reason that the cerebellum may not be visible to EEG or MEG may arise from some historical methodological limitations that have since been overcome. EEG and MEG studies employing event-related averaging, inherently optimized to distinguish phase-locked evoked activity, have rarely suggested cerebellum activation. Even experiments employing invasive recordings in animal cerebellum only occasionally report event-related potentials (ERPs) (e.g., Rowland and Jaeger, 2008); the vast majority of such studies instead report modulations of oscillatory activity (see de Zeeuw et al. (2008) for a review).

This suggests that the cerebellum may primarily exhibit oscillatory modulations that may not necessarily be phase-locked. Indeed, the classic experiments of Adrian (1934) [cat, 40–300 ​Hz], Dow (1938) [cat, 150–250 ​Hz], Ten Cate and Wiggers (1942) [cat, 50–230 ​Hz], and Pellet (1967) [guinea pig, 200–400 ​Hz] all demonstrated high-frequency oscillatory activity in the cerebellum. Niedermeyer and Uematsu (1974) observed low-frequency oscillations (1.5–6 ​Hz) in three human Lennox-Gastaut syndrome patients implanted with cerebellar electrodes in an experimental attempt at stimulation treatment. de Solages et al. (2008) showed that the Purkinje cell layer produces 200 ​Hz oscillations in Wistar rats, which seem to entrain unit firing; high-frequency LFPs in the molecular and granule cell layers were far less pronounced. More recently, Cheron and Cheron (2018) found that stimulation of the inferior olive in mice induced high-frequency oscillations (350 ​Hz) in the cerebellum. Intracranial recordings from the human cerebellum are exceedingly rare, but Dalal et al. (2013) reviewed the sparse literature describing them, and re-analysed some key historical intracranial recordings of the human cerebellum, three published in Russian (Irger et al., 1949a, 1949b; 1951) and one in French (Rétif, 1964). In the studies by Irger et al., the human cerebellum exhibited spontaneous oscillations in the beta band range (15–30 ​Hz) and in both the low-gamma (35–50 ​Hz) and high-gamma (80–100 ​Hz) ranges, The recordings from Rétif (1964) furthermore revealed evidence of 250 ​Hz oscillations. The few intracranial recordings from the human cerebellum thus seem to correspond to the animal literature.

Perhaps the neuronal mechanisms or morphology of the cerebellum preclude robust production of phase-locked evoked responses, which would have given the impression that the cerebellum is silent to scalp EEG/MEG for several years until the revival of non-phase-locked analyses using time-frequency techniques. Indeed, more compelling MEG findings of cerebellar activity came about after techniques to perform time-frequency analysis in source space became more widely available (e.g., Gross et al., 2002; Dalal et al., 2008; Pollok et al., 2008; Schnitzler et al., 2009; Kennedy et al., 2011).

Sensor coverage may also be a factor. The traditional 10/20 EEG system and even state-of-the-art high-density electrode caps as well as most whole-head MEG systems may simply not provide sufficient spatial sampling over the regions where cerebellar signals may project, e.g., the top of the neck. This problem can be partially overcome using low-tech solutions, such as thoughtful placement of subjects in traditional MEG sensor arrays (perhaps with the head tilted more forward than usual for better cerebellar coverage at the expense of frontal coverage as in Hashimoto et al. (2003), or the use of additional free electrodes further down the neck to supplement an EEG cap. With the advent of on-scalp MEG techniques such as optically pumped magnetometers (OPMs) (Boto et al., 2017) and high critical temperature (high-*Tc*) SQUIDs (Pfeiffer et al., 2019), it is also becoming possible to place sensors freely, and thus place them as close as possible to the cerebellum, on the back of the head or possibly even into the mouth to approach it from the other side.

Source localization attempts have traditionally assumed a spherical head model fit to cerebral cortex, perhaps resulting in a poor fit with cerebellar cortex. Implementations of realistic head models usually neglect the cerebellum, either removing it completely or including it within the same compartment as cerebral cortex. Additionally, techniques that assume sources to be oriented orthogonally to the cortical surface may need refinement for the cerebellum, as the cerebellum is less easily segmented. The cerebellum, due to its different morphology as well as its separation of cerebral cortex by thick dura mater (the cerebellar tentorium), may ultimately profit from realistic models that specifically take into account its electrical properties. Ramon et al. (2014) showed through simulation that head models that did not model the dura mater would overestimate the corresponding electric potential in EEG. Likewise, in MEG, given that secondary currents have been shown to contribute to the measured signal (Stenroos et al., 2014), this suggests that some improvement in MEG source localization may be achieved by modelling the cerebellar tentorium carefully.

Finally, it has long been speculated that the especially fine folding of the cerebellum may lead to substantial cancellation of signals recorded at the distance of MEG or EEG, due to the likelihood that sources on opposite sulci (with therefore opposing current flow) are simultaneously active. Recently, however, Samuelsson et al. (2020) quantified the degree of signal cancellation that can be expected, based on a boundary element model created of the cerebellum from a very high resolution ex vivo MRI (190 ​μm voxel size at 9.4T). Their simulations estimate that the cerebellar signal should be attenuated only 30–60% on average relative to cerebral cortex, suggesting that the final signal strength of much of the cerebellum should still be well within the sensitivity limits of MEG and EEG.

## 3 Previous reports and illustrative examples

As mentioned earlier, the introduction of time-frequency analyses greatly increased the number of published findings on cerebellar activity stemming from mainly MEG recordings and some EEG recordings. We again emphasize that this spectral information would not be extractable using fMRI. Here, we will go through some of them in greater detail. We do not intend this to be a systematic review that includes all EEG and MEG papers that have been published on cerebellar activation, but rather a set of illustrative examples (Table 1) showcasing that MEG and EEG are not blind to the cerebellum.

**Table 1:** Studies reporting cerebellar findings using MEG or EEG sorted by domain, subject group, type of response and method for source localization. ∗Information obtained from personal communication.

Authors and year	Modality	Domain	Subject group	Response	Source localization	Head model
Gross et al. (2002)	MEG	Motor	Neurotypical (*N* ​= ​9)	Long-range EMG connectivity	Beamformer (DICS)	Single shell head model (Nolte, 2003), based on individual MRs∗
Timmermann et al. (2003)	MEG	Motor	Parkinson’s Disease patients (*N* ​= ​6)	Long-range EMG connectivity	Beamformer (DICS)	Single shell head model (Nolte, 2003), based on individual MRs
Pollok et al. (2005)	MEG	Motor	Neurotypical (*N* ​= ​10)	Long-range EMG connectivity	Beamformer (DICS)	Single shell head model (Nolte, 2003), based on individual MRs
Jerbi et al. (2007)	MEG	Motor	Neurotypical (*N* ​= ​15)	Long-range connectivity	Minimum-norm estimate	Single sphere head models based on individual MRs∗
Dalal et al. (2008)	MEG	Motor	Neurotypical (*N* ​= ​12)	Oscillations. ECoG used to validate results on epilepsy patients (*N* ​= ​2)	Beamformer	Multiple spheres model (Huang et al., 1999), based on individual MRs
Pollok et al. (2008)	MEG	Motor	Neurotypical (*N* ​= ​11)	Long-range EMG connectivity	Beamformer (DICS)	Single shell head model (Nolte, 2003), based on individual MRs
Schnitzler et al. (2009)	MEG	Motor	Essential Tremor patients (*N* ​= ​8)	Long-range EMG connectivity	Beamformer (DICS)	Single shell head model (Nolte, 2003), based on individual MRs
Wilson et al. (2010)	MEG	Motor	Neurotypical children and adolescents (*N* ​= ​10)	Oscillations (beta band)	Beamformer (DICS)	Single shell head model, based on individual MRs∗
Marty et al. (2018)	MEG	Motor	Neurotypical (*N* ​= ​11)	Long-range connectivity	Beamformer	Single shell head model (Gramfort et al., 2013), based on individual MRs
Torres and Beardsley (2019)	EEG	Motor	Neurotypical (*N* ​= ​15)	Event-related potentials	Minimum-norm estimate	Three-layered Boundary Element Method (OpenMEEG; Gramfort et al., 2010), based on template brain with individual electrode locations
Reyes et al. (2005)	EEG	Audition	Neurotypical (*N* ​= ​9)	Steady-state response	Minimum-norm estimate (LORETA)	Three-layered Boundary Element Method (Curry 4.5 Neuroscan Labs Inc., El Paso, TX), based on template brain with individual electrode locations
Herrojo Ruiz et al. (2017)	MEG	Audition	Neurotypical (*N* ​= ​21)	Oscillations (theta and beta bands)	Dipole fitting	Single shell head model (Gramfort et al., 2013), based on individual MRs
Cao et al. (2017)	MEG	Audition	Neurotypical (*N* ​= ​10)	TMS and event-related fields	Minimum-norm estimate (eLORETA)	Single shell head model (Nolte, 2003), based on individual MRs
Tesche and Karhu (1997)	MEG	Somatosensation	Neurotypical (*N* ​= ​4)	Event-related fields.	Dipole time course estimation	Single shell head model (Hämäläinen and Sarvas, 1989), based on individual MRs
Tesche and Karhu (2000)	MEG	Somatosensation	Neurotypical (*N* ​= ​9)	Event-related fields and oscillations.	Dipole time course estimation	Single shell head model (Hämäläinen and Sarvas, 1989), based on individual MRs
Hashimoto et al. (2003)	MEG	Somatosensation	Neurotypical (*N* ​= ​12)	Event-related fields	Beamformer	Single sphere head model, based on individual MRs
Andersen and Lundqvist (2019)	MEG	Somatosensation	Neurotypical (*N* ​= ​20)	Oscillations (theta and beta bands)	Beamformer (DICS)	Single shell head model (Nolte, 2003), based on individual MRs
Jousmäki et al. (1996)	MEG	Visuomotor	Neurotypical (*N* ​= ​8)	Event-related fields	Dipole fitting	Single sphere head model, based on individual MRs
Bourguignon et al. (2013)	MEG	Visuomotor	Neurotypical (*N* ​= ​10)	Long-range connectivity	Beamformer (DICS)	Not indicated
Cebolla et al. (2016)	EEG	Visuomotor	Astronauts in space and on Earth (*N* ​= ​5)	Oscillations (alpha band)	Minimum-norm estimate (swLORETA)	Boundary element method, layers not specified, based on template MR
Guggisberg et al. (2008)	MEG	Cognition	Neurotypical (*N* ​= ​10)	Oscillations (Gamma)	Beamformer	Multiple spheres head model, based on individual MRs
Guggisberg et al. (2011)	MEG	Cognition	Neurotypical (*N* ​= ​11)	Oscillations (Gamma)	Beamformer	Multiple spheres head model, based on individual MRs
Styliadis et al. (2015)	MEG	Emotion	Neurotypical (*N* ​= ​12)	Oscillations (Gamma)	Beamformer (SAM)	Multiple spheres head model, based on individual MRs
Niedermeyer and Uematsu (1974)	EEG	Epilepsy	Epileptic patients (N ​= ​3, ages 16, 18, 34)	Ictal and apparently normal sleep/drowsiness waveforms	Simultaneous intracranial EEG	None
Mohamed et al. (2011)	MEG	Epilepsy	Epileptic child (*N* ​= ​1)	Ictal and validated with iEEG	Beamformer	Not indicated
Lascano et al. (2013)	EEG	Epilepsy	Epileptic child (*N* ​= ​1)	Intal and interictal.	Minimum-norm-estimate (LAURA)	Spherical Model with Anatomical Constraints (Spinelli et al., 2000) with individual MR
Elshoff et al. (2013)	EEG	Epilepsy	Epileptic children (*N* ​= ​11)	Ictal	Beamformer (DICS)	Five-concentric-spheres model with a single sphere for each layer corresponding to the white matter, grey matter, cerebral spinal fluid (CSF), skull and skin, based on individual MRs
Kujala et al. (2007)	MEG	Reading	Neurotypical (*N* ​= ​9)	Oscillations (alpha) and phase coupling	Beamformer (DICS)	Single-layer Boundary Element Method, based on individual MRs∗
Brookes et al. (2011)	MEG	Resting state	Neurotypical (*N* ​= ​10)	Independent components	Beamformer	Not indicated
Wibral et al. (2011)	MEG	Auditory memory	Neurotypical (*N* ​= ​22)	Oscillations (Gamma) and transfer entropy	Beamformer	Single shell head model (Nolte, 2003), based on individual MRs
Lin et al. (2019)	Optically pumped magnetometers	Air-puffs to the eyes	Neurotypical (*N* ​= ​3)	Event-related fields and oscillations	Dipole fitting (event-related fields) and beamformer (oscillations)	Single shell head model (Nolte, 2003), based on individual MRs

### 3.1 Motor tasks

Gross et al. (2002), based on the application of Dynamic Imaging of Coherent Sources (DICS; Gross et al., 2001), found coherence between electromyography (EMG) resulting from a sinusoidal movement and MEG activity in the contralateral sensorimotor cortex, i.e., corticomuscular coherence. They then localized the brain areas coherently oscillating with sensorimotor cortex, among which they found ipsilateral cerebellum, thalamus and premotor cortex (PMC) engaged in a feedback loop oscillating at a rhythm of 8–10 ​Hz, corresponding to natural discontinuities in movement (Vallbo and Wessberg, 1993).

**Fig. 2:**
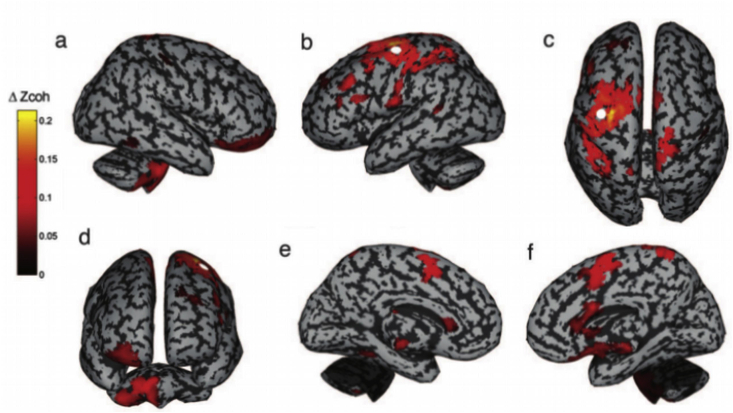
**Strength of task-based coherence with primary cortex as a reference:** subjects were to counteract the unpredictable movements of a cube rotating around its centre by moving a trackball. The kinematics of the trackball movement were registered and its coupling to the neural time series were estimated, using task-related Z-transformed coherence with M1 activity (white dot) as an outcome measure (ΔZcoh), showing coherence with the cerebellum. Figure from Jerbi et al. (2007).

Pollok et al. (2005), also using DICS, extended the network to also include supplementary motor area (SMA) and posterior parietal cortex (PPC), while Pollok et al. (2008) showed that anticipated movements were related to an increase in coupling directed from cerebellar to thalamic to parietal areas, i.e., cerebellum to cerebrum, whereas non-anticipated movements were related to an increase in coupling direction from parietal areas to cerebellar areas, i.e., cerebrum to cerebellum. Pollok et al. interpreted these two differentially directed couplings as anticipatory motor control and mismatch detection, respectively. Jerbi et al. (2007) (Fig. 2) found that these cerebellar couplings to motor cortex also encode the speed with which hand movements are made. All these studies take advantage of using a peripheral EMG signal to reduce the space of potential coherence tests to be made, by focusing the brain-to-brain coherence tests on only the brain region that included the greatest corticomuscular brain-muscular coherence. Furthermore, they used simple motor tasks in which the role of the cerebellum is undisputed and used individual MRs to create realistic head models.

**Fig. 3:**
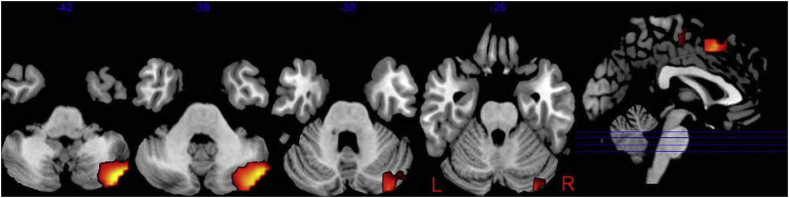
**Pre-movement beta activation in cerebellar cortices.** Beta activation in ipsilateral cerebellar cortices following a flexion-extension movement. The maximum is in the inferior portions of ipsilateral cerebellum crus II. This figure is adapted from Wilson et al., (2010) with permission.

Wilson et al. (2010) (Fig. 3) found cerebellar activity in the beta band (15–30 ​Hz) before and after movements. Finally, Dalal et al. (2008) furthermore found cerebellar activity in high gamma frequencies (>65 ​Hz) when subjects performed finger movements. Essentially, the same task was used, but localization was based on the whole brain rather than on those coherent with the region showing the greatest corticomuscular coherence.

Taken together, these studies provide consistent evidence that cerebellar activity can be detected in MEG when simple motor movements are performed. Finally, a recent EEG study has used distributed models to reconstruct phase-locked activity (Torres and Beardsley, 2019) related to simple flexions of the wrists. It remains to be seen whether the same could be done with MEG, which would be very interesting, especially since these methods are very simple to apply.

In addition to detection of cerebellar activity in healthy participants, cerebellar activity has also been detected in patients with dysfunctional networks or motor pathologies.

Using DICS, and the same general methods that Gross et al. (2002) used, Timmermann et al. (2003) found oscillatory coherence between the EMG of the hand tremor of six Parkinson patients and their contralateral M1. Similar to Gross et al. (2002), they found evidence of coherence between contralateral M1 and ipsilateral cerebellum. Schnitzler et al. (2009) similarly found oscillatory coherence between the EMG of the hand and the contralateral M1 in eight patients with Essential Tremor. Again using DICS, they also found coherence between M1 and ipsilateral cerebellum. Similar results have been found for the tremor related to Wilson’s disease (Südmeyer et al., 2006).

### 3.2 Somatosensation

MEG-based evidence for the cerebellum’s involvement in pure somatosensation was reported earlier than the evidence for its involvement in motor control. Tesche and Karhu (1997) found that median nerve stimulation elicited cerebellar event-related responses, contrary to the motor studies above where only long-range EMG connectivity was reported. Furthermore, they found (2000) that omissions of otherwise expected somatosensory stimulations elicited oscillatory activity following the time point when the stimulation should have happened, and that cerebellar oscillatory activity increased again before the following anticipated stimulation. Note that activity was not strictly speaking *localized* to the cerebellum in these studies, rather they estimated time courses for cerebellar sources given the assumption that there were sources there in the first place. As they also acknowledge, when time courses are estimated like this, it is possible that sources include activity generated at sources adjacent to the assumed source. Hashimoto et al. (2003), however, used a beamformer technique to localize median nerve stimulation evoked responses to the cerebellum. This study will be discussed more in-depth in a later section (Section 4) due to the importance of sensor coverage that it highlights.

In addition to EMG, the kinematic signals of body movements have also been found to couple with the cerebellum in various contexts, a phenomenon referred to as *corticokinematic coherence* (CKC). For example, Marty et al. (2018) found that cerebellar activity entrains to the speed and kinematics of finger movements. A recent review of the relevant literature argues that CKC reflects proprioceptive spinocortical afferent signals, in contrast to CMC, which reflects corticospinal efferent signals (Bourguignon et al., 2019). The strategy here is very similar to using EMG as a peripheral signal to reduce the coherence source space (Gross et al., 2002).

**Fig. 4:**
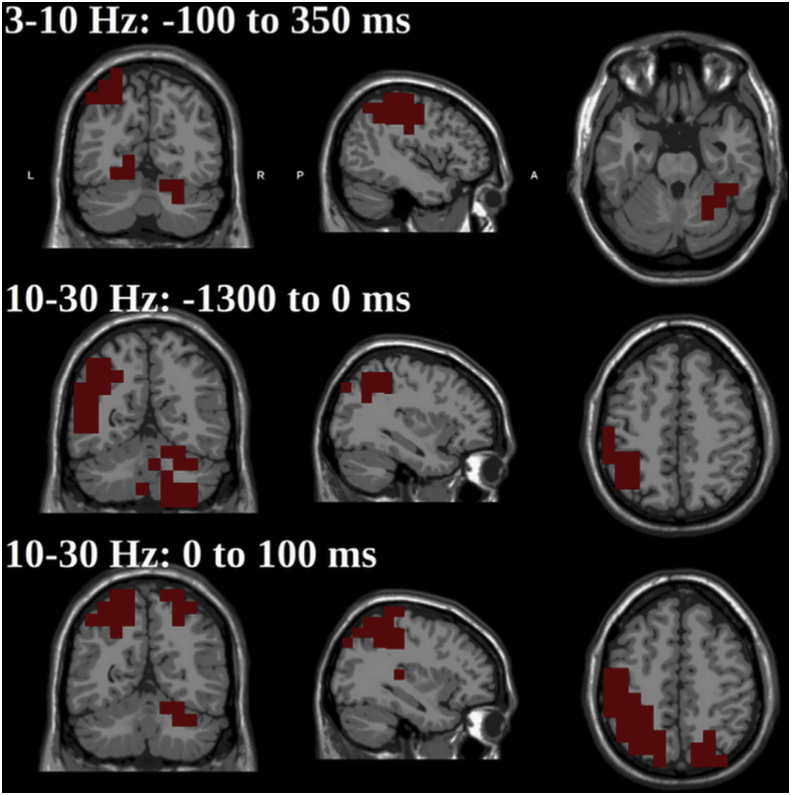
**Differences in cerebellar activation between expected and unexpected stimulations.** Subjects had their right index finger stimulated rhythmically (every 3 ​s). Every now and then a stimulation was omitted. The contrasts shown here indicate brain regions exhibiting significantly more power for *repeated stimulations* (a stimulation following another stimulation) than for *first stimulations* (a stimulation following an omission), where 0 ​ms refers to stimulation onset. This figure is adapted from Andersen and Lundqvist (2019) under the CC BY 4.0 licence.

Not relying on coupling, Andersen and Lundqvist (2019) (Fig. 4), using DICS, localized cerebellar oscillatory activity related to updating and maintaining expectations about somatosensation, ipsilateral to the stimulated hand in the theta and beta bands similar to the auditory study of Herrojo Ruiz et al. (2017) discussed below. These two studies indicate that low-frequency cerebellar oscillations may be related to updating and maintaining expectations. An important difference between the study of Andersen and Lundqvist (2019) and the motor studies discussed above is that they are indirectly dependent on a peripheral reference signal usually EMG or kinematics of hand movement. In most of the motor studies cited, first coherence between an external reference, e.g. EMG, and M1 activity is established, and second the coherence between M1 and other areas are investigated. Andersen and Lundqvist (2019), although also using DICS, instead investigated the whole brain using the power maps output by DICS. This suggests that, using state-of-the-art MEG and source reconstruction methods, it is possible to retrieve cerebellar signals.

### 3.3 Audition

Using MEG, Riaz et al. (2017), investigated auditory feedback related to motor movements (playing the piano). They provided feedback that was either expected (related to the movement) or unexpected (unrelated to the movement). When unexpected feedback was received, they found differences in the theta (3–7 ​Hz) and beta bands (15–30 ​Hz) to when expected feedback was received. Subsequently, they used common spatial pattern (CSP) analysis (Blankertz et al., 2008) to find the patterns of activity that were most strongly related to the differences between expected and unexpected feedback and fitted dipoles to these patterns that revealed the cerebellum as a generator. Also using MEG, Cao et al. (2017), found that attenuation of self-generated tones, as indicated by the decrease of the auditory fields, was decreased when cerebellar activity was disrupted with TMS. They found that the cerebellar vermis was more active during actual attenuation, i.e. during the sham condition of the TMS. The source reconstruction was based on event-related fields (ERFs) using the eLORETA algorithm.

Using EEG, Reyes et al. (2005), found evidence of cerebellar involvement in the so-called 40 ​Hz auditory steady-state response (ASSR). The 40 ​Hz ASSR is an oscillation arising when tones are amplitude-modulated at a frequency of 40 ​Hz. Using the LORETA algorithm, they localized activity in the left cerebellum both when reconstructing the activity weighted and unweighted by an independent PET scan.

### 3.4 Visuomotor

Jousmäki et al. (1996) had subjects perform horizontal saccades every 3 ​s. Using a non-linear least squares fit, they fitted a two-dipole model in eight subjects, each resulting in one dipole localized to cerebellum and one localized to the posterior parietal cortex. These revealed evoked responses ~170 ​ms after the onset of the saccade. In contrast to the studies of Tesche and Karhu (1997, 2000), these dipole fits represent a source localization and not estimates of time courses. Bourguignon et al. (2013) had subjects observe an experimenter moving his finger rhythmically (3 ​Hz). Using methods similar to Gross et al. (2001), DICS, reducing the coherence source space, they found that the motor cortices of subjects were coherently oscillating with the oscillating 3 ​Hz movement of the experimenter. Furthermore, they found that the primary motor cortex was coherently oscillating with cerebellum and V3 also at 3 ​Hz.

Using EEG in a visuomotor task, Cebolla et al. (2016) compared the alpha-mu (~8–12 ​Hz) oscillations in astronauts when they were either in a weightless state (in space) or on Earth. They found greater desynchronization of the mu rhythms when the astronauts were visually attending to target stimuli when the astronauts were in space compared to when they were on Earth. Using a LORETA-style algorithm, cerebellum was revealed to contribute to this difference, possibly reflecting activation necessary for postural stabilization.

### 3.5 Cognition

High-gamma oscillations (~60–180 ​Hz) in the cerebellum have also been implicated in decision making and introspection about decisions, perception and movement. Guggisberg et al. (2008) found high-gamma oscillations in the cerebellum when participants make decisions related to numerical representation, explicit memory and self-representation. They specifically found that the left cerebellar hemisphere, together with the inferior parietal lobule, were the key structures involved with internally cued decisions. Guggisberg et al. (2011) found that the cerebellum was part of a network activated when participants were asked to introspect the timing of three kinds of events: phoneme perception, their own response decision, or the movement manifesting that decision. Both of these studies made use of the time-frequency beamformer technique introduced by Dalal et al. (2008), together with group statistics based on statistical non-parametric mapping (SnPM; Singh et al., 2003). Finally, Styliadis et al. (2015) using MEG found that oscillations (60–100 ​Hz) reflecting emotional arousal, emotional valence and their interaction were localized to distinct areas of the cerebellum using an LCMV (linearly constrained minimum-variance) beamformer. Moreover, they followed a temporal hierarchy with arousal being processed before valence.

### 3.6 Epilepsy

A few reports of cerebellar activity in epilepsy patients also exist. Niedermeyer and Uematsu (1974) presented three cases of epilepsy patients who were candidates for an experimental therapy of the time involving cerebellar stimulation. They exceptionally had recordings from a patient implanted with depth electrodes directly in the cerebellum simultaneously with scalp EEG, which showed that the cerebellum’s activity, including apparently normal sleep spindles as well as seizure activity, could be shown in both the invasive and non-invasive recording leads. Mohamed et al. (2011) found, using an LCMV beamformer on MEG broadband activity (25–100 ​Hz), cerebellar activity 14 ​s after ictal onset in the motor cortex in a four-year old boy. They discuss the possibility that the delayed cerebellar activity may play a modulatory role in seizure termination. Lascano et al. (2013), however, found evidence of a cerebellar lesion as the primary seizure generator in a 14-month old girl from high-density scalp EEG, which was subsequently confirmed by intracranial EEG performed immediately prior to surgical resection as well as freedom from seizures post-operatively. Finally, Elshoff et al. (2013) tested sources underlying the frequency spectrum in EEG epochs of 10 ​s recorded during seizures. Using DICS, they found cerebellar activity in 5 out of 11 patients. The patients were between 1 and 19 years old (mean age: 9.6 years).

### 3.7 Resting state and network investigations

There is a great amount of resting state studies in the fMRI literature (van den Heuvel and Hulshoff Pol, 2010), and the cerebellum has also been found to be part of the so-called default mode network (Bernard et al., 2012; Sang et al., 2012). Brookes et al. (2011) used a combination of beamforming and independent component analysis (ICA) to retrieve the components making up the default mode network. The components they found showed a great overlap with the components found in the fMRI literature. The cerebellar components specifically were found in the beta band range (13–30 ​Hz). Wibral et al. (2011) investigated auditory short-term memory and found cerebro-cerebellar connections in the gamma band (60–120 ​Hz) using transfer entropy and DICS beamforming. Finally, Kujala et al. (2007) investigated phase coupling in a reading task and found cerebro-cerebellar couplings in the alpha band (8–13 ​Hz) during reading using a DICS beamformer.

### 3.8 Summary

Taken together, these studies show that cerebellar activity can, under certain circumstances, be detected with MEG and EEG. Many of the studies rely on an external reference, e.g., movement and observed movement, for establishing coherence between areas, and it is the coherence between oscillations that is detected rather than a standard task-related source activation. The studies of Jousmäki et al. (1996), Hashimoto et al. (2003), Cao et al. (2017) and Torres and Beardsley (2019) are also noteworthy for their detection of event-related fields in the cerebellum, where most other studies detect oscillatory responses.

## 4 How can we enhance our ability to monitor cerebellum with MEG?

In this section, we will cover methodological approaches that can enhance the chances of detecting cerebellar activity with MEG. We describe approaches that have successfully been used to detect cerebellar activity and discuss further promising strategies.

### 4.1 Optimizing design (superficial targets and initial localization)

The signal of more anterior parts of the cerebellum is going to be comparatively small, purely due to the distance to the MEG sensors. If possible, one could aim to target cerebellar areas that are superficial, relatively speaking. This would of course require that studies based on other modalities had implicated the specific cerebellar region. For inspiration, one could look at the detailed functional mapping of King et al. (2019). A related strategy would be to use a paradigm that robustly elicits a cerebellar response that can also be robustly localized. Using such a paradigm, a cerebellar source could be initially localized and thereafter its time course could be estimated for more subtle manipulations and variations of the localization paradigm. The question is though whether such a paradigm exists. A possible candidate might be the eye-blink conditioning paradigm. In eye-blink conditioning, performing an eye-blink is conditioned to the onset of tone (Conditioned Stimulus) which is followed by an air-puff to the eye (Unconditioned Stimulus). This conditioned response is dependent on an intact cerebellum (McCormick and Thompson, 1984). Kirsch et al. (2003) found evidence of cerebellum’s involvement in this response using MEG. Note however that their strategy is similar to that of Tesche and Karhu (1997, 2000) where they estimate the time course of assumed cerebellar sources. Recently, Lin et al. (2019) showed promising evidence of using the air-puff paradigm. This will be discussed further in section 4.7.

### 4.2 Coverage of MEG sensor array or EEG coverage

A recent study of Todd et al. (2018) extended the 10–20 layout with extra electrodes below electrode *Oz*. They very interestingly found that these “cerebellar” electrodes picked up high-frequency oscillations (>100 ​Hz) that were unique to these electrodes and not found on the occipital electrodes above nor the splenius muscle electrodes below. This highlights the importance of actually covering the cerebellum such that signal can be picked up in the first place.

**Fig. 5:**
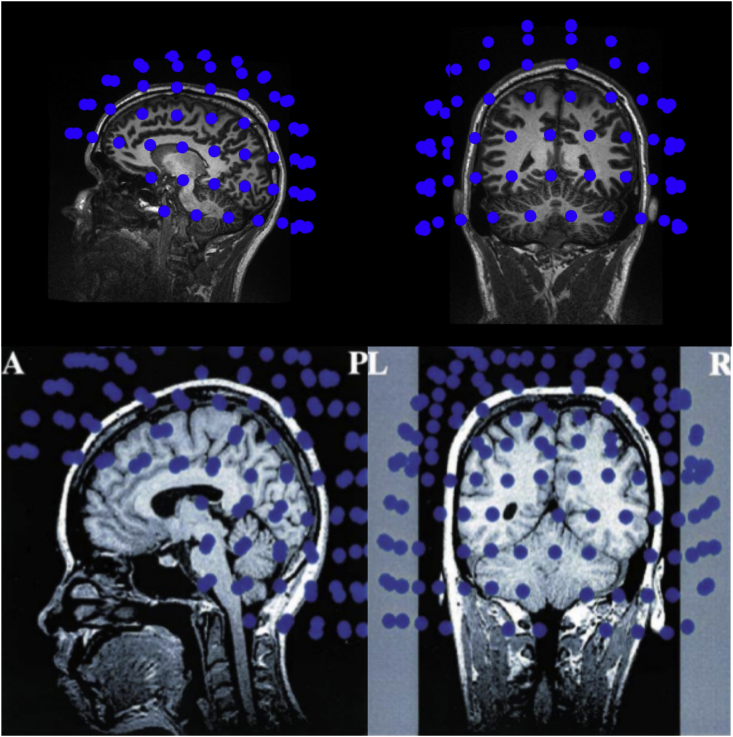
**Tilting the head to obtain better sensor coverage of the cerebellum.** The upper panel shows a typical head placement in a modern MEG system, the Neuromag Triux, with its 102 sensor locations depicted in blue. While the cerebellum is partially covered with this positioning, tilting the head backwards relative to the sensor array may provide a more complete coverage of the cerebellum. Hashimoto et al. (2003) demonstrates such a positioning with a 160-channel Yokogawa MEG system, as seen in the lower panel, reproduced with permission (A ​= ​Anterior, P=Posterior, L ​= ​Left, R ​= ​Right).

Hashimoto et al. (2003) investigated somatosensory fields evoked by median nerve stimulation using the Yokogawa MEGVISION with 160 axial gradiometers. Using a beamformer method (Sekihara et al., 2001), they were able to reconstruct fields as arising from the medial part of the cerebellum. As can be seen in Fig. 5, sensor coverage extended below the cerebellum, including the upper cervical spine. This seems to have been done by having subjects tilting their heads forwards relative to the helmet. This meant that some frontal coverage was sacrificed at the expense of being able to sample the cerebellum. This is a simple strategy that may be highly beneficial. As seen in the bottom row of Fig. 5, the cerebellum is not fully covered when the subject does not tilt their head.

### 4.3 Careful artefact removal

Cerebellar responses are susceptible to masking by or confounding with neck muscle EMG. It is therefore recommendable to record EMG from the major neck muscles. Especially, Minimum-Norm-Estimate-like and dipole fitting source reconstructions (Hämäläinen and Ilmoniemi, 1994) would benefit from this, since these will allocate all magnetic fields recorded by the sensors to the assumed source space. If the source space includes cerebellum, and neck muscle activity is not removed before source reconstruction, the neck muscle activity is likely to be source reconstructed as spuriously arising from the cerebellum. Even in the presence of artefacts, beamformer methods will be useful since these reconstruct source activity independently at each assumed source location. This is done by creating a spatial filter that minimises contributions from other sources, brain and noise alike.

### 4.4 Long-range coupling

A successful strategy for localizing cerebellar activity has been to localize it based on its coherence with a “far-away” signal such as the EMG or kinematic signals of the foot or the hand (Bourguignon et al., 2019) as discussed in Section 3. Using long-range coupling adds a level of trustworthiness to the connectivity assessments, since short-range connectivity assessments have many interpretational pitfalls (Bastos and Schoffelen, 2016; Schoffelen and Gross, 2009). The paradigms of Gross’s and Jerbi’s groups have been very successful in applying this strategy (see Section 3). The kinds of paradigms that can be run with these kinds of strategies might be limited to sensory and motor paradigms, however.

### 4.5 Reducing neocortical activity using cortical signal suppression

Samuelsson and Hämäläinen (2019) have developed the method of cortical signal suppression (CSS). The overall idea of this method is based on using unique features respectively of planar gradiometers and of magnetometers, as in the Neuromag system. Colloquially said, planar gradiometers are “near-sighted”, being maximally sensitive to signals arising from the cerebral cortex, whereas magnetometers are also sensitive to signals from beyond the cerebral cortex. By projecting out the signal shared between the magnetometers and planar gradiometers from the signal of the magnetometers alone, one can obtain a magnetometer signal that uniquely represents non-cerebral cortex. Applying this method to the auditory steady state response (ASSR), they were able to decrease the ASSR signal arising from cerebral cortex by 97%, while in turn increasing the ASSR signal arising subcortically by 10%. The method has not been applied to investigate cerebellar activity yet. Another interesting aspect about this method is that it does not require any special data acquisition procedures. Thus, already acquired data sets are likely to benefit from re-analysis using CSS if cerebellum or sub-cortical sources are expected.

### 4.6 Improving anatomical models of cerebellum

In beamformer applications, the orientations of the sources are normally not included in the source model. Instead, the direction that maximizes the beamformer’s output SNR is typically chosen as the source orientation, determined through an optimization based on singular value decomposition (Sekihara et al., 2004). However, Hillebrand and Barnes (2003) found that the signal of the beamformer could be improved if anatomical constraints were introduced, such that sources were correctly oriented in the source model. The improvement in signal, however, is critically dependent on the co-registration error between MEG and MRI and the precision of the estimate of the orientation of the sources. Hillebrand and Barnes (2003) concluded that these errors need to be smaller than 2 ​mm and 10° respectively for these anatomical constraints. Regarding the co-registration error, several different strategies have been developed to reduce the error to less than 2 ​mm, e.g., photogrammetry (Clausner et al., 2017), structured-light scanner (Zetter et al., 2019; Homölle and Oostenveld, 2019), and head casts (Meyer et al., 2017).

Regarding the estimation of source orientations, the typical anatomical constraint for MEG is to assume sources are orthogonal to the cortical surface extracted from anatomical T1 MRI scans. However, high-quality cortical surface extraction from 1.5T or 3T MRI is less tractable for the cerebellar cortex due to its thinness, leading to the unfortunate consequence that most available source analysis pipelines that depend on cortical surface information simply drop the cerebellum from the source space entirely. 7T MRI can yield sufficient resolution for reasonable extraction of the cerebellar cortical surface (Boillat et al., 2018). Samuelsson et al. (2020) emphasizes the importance of high-resolution cerebellar surface models, since they show that standard segmentations of the cerebellum give rise to an overestimation of the net cerebellar signal due to underestimation of signal cancellation, which can be avoided with high-resolution models. The high-resolution model also shows that more signal cancellation occurs in the cerebellum than in cerebral cortex, but that nonetheless cerebellar activity should be detectable using MEG and EEG. As an alternative to surface extraction from high-resolution scans, it has been suggested that neural fibre orientations may be derived from customized diffusion-weighted MRI (DWI) sequences at 3T; preliminary investigations suggest that this method can help distinguish activations of the visual cortex from the cerebellum (Dalal et al., 2018). It must be emphasized that invasive electrophysiological recordings of the cerebellum alongside MEG and/or EEG at a distance that provide actual information about the magnitude of signal cancellation in the cerebellum are still missing, but the modelling of Samuelsson et al. (2020) shows that signal cancellation in the cerebellum is not likely to make MEG and EEG recordings of cerebellum infeasible.

### 4.7 Speculation for the future - on scalp MEG

Several technologies are being developed where the ambition is to create whole-head arrays of on-scalp, or nearly on-scalp, MEG sensors. One alternative is to use high-*Tc* SQUIDs (Pfeiffer et al., 2019; Öisjöen et al., 2012). Successful recordings of somatosensory and auditory fields have been made using these (Andersen et al. 2017, 2019; Pfeiffer et al., 2019). At present, arrays of up to 7 high-*Tc* SQUID magnetometers have been created. These can virtually be placed on the scalp (<1 ​mm). Another alternative, optically pumped magnetometers (OPMs), are already commercially available for assembly into small-scale systems suitable for MEG. Recordings with 20 OPMs have been conducted and can also be placed close to the scalp ~6.5 ​mm (Borna et al., 2017; Boto et al., 2017). Since the pickup coil size of the magnetometers can be made smaller when moving towards the scalp, the spatial resolution will increase. This allows for sampling magnetic fields related to more focal brain activity than could be obtained with state-of-the-art MEG. As discussed earlier, one oft-mentioned reason that the cerebellum is purportedly not visible to MEG is that it is more finely folded than the cerebral cortex, resulting in signal cancellation. With finer spatial resolution, the problem of signal cancellation may be mitigated. Interestingly, the aforementioned Yokogawa system (Hashimoto et al., 2003) had a smaller pickup area 189 mm2 than current CTF-systems (254 mm2) and Neuromag systems (441 mm2**)**. In comparison, the size of the pickup coils in high-Tc SQUIDs is 81 mm2 (Andersen et al. 2017, 2019; Pfeiffer et al., 2019), and the equivalent pickup area modern OPMs is even smaller; for example, the surface area of the vapour cell of the QuSpin QZFM OPM is only 9 mm2 (Osborne et al., 2018). These new technologies are likely to usher in a new exciting age for recordings of cerebellar MEG. In fact, a report already exists of OPMs being used to record evoked fields arising from the cerebellum (Lin et al., 2019). It furthermore seems likely that on-scalp technologies may be used to recover evoked responses from the cerebellum when doing classical median nerve stimulation as Hashimoto et al. (2003) did. On-scalp MEG may also improve SNR for high-frequency oscillations (Krishnaswamy et al., 2017) since it samples brain activity more sparsely than conventional MEG that samples the brain from a distance. It is important to note how bandwidth and sensitivity are inversely related in OPMs (Tierney et al., 2019). This has the implication that one cannot sample low- and high-frequency activity at the same time using the same sensor. On the other hand, some types of OPMs can be individually tuned such that their bandwidth is appropriate for picking up the relevant activity (Jiménez-Martínez et al., 2012). Bandwidth for high-*Tc* SQUIDs however is the same as for state-of-the MEG systems.

When designing the arrays, it is important that sensors are included in lower positions than the fixed low-*Tc* SQUID arrays currently include (Fig. 5). It is also feasible to build flexible arrays with both high-*Tc* SQUIDs (Riaz et al., 2017) and OPMs (Boto et al., 2019) meaning that one may be able to position the sensors according to what is optimal for one’s present paradigm. Finally, using multiple layers of sensors may allow for better separation of cerebral cortex and deep sources akin to the use of reference sensors in the CTF (CTF MEG International Services LP. Coquitlam, BC, Canada) MEG systems. The reference sensors are used for noise cancellation (Boto et al., 2017), but could potentially be used to suppress cortical signals as well, related to the cortical signal suppression technique discussed above (Samuelsson et al., 2019).

### 4.8 Source localization of cerebellar MEG and cerebellar EEG respectively

There are well-known differences between MEG and EEG in terms of source localization and sensitivity. In the cerebral cortex, the sensitivity profiles of MEG and EEG differ in terms of MEG being insensitive to gyral activations - it may be thought that similar differences arise in the cerebellum, but due to the fine folding of the cerebellum, sulci and gyri are not well defined. As MEG source localization is less sensitive to inaccuracies in the head model than EEG source localization is, then, given identical head models, MEG source localization will thus likely be more precise and accurate than EEG source localization. Close approximations may work well for MEG, but less so for EEG.

Thus, careful attention especially needs to be given to EEG head models for them to be useful in corroborating cerebellar source localization. Careful attention to MEG head models will also be likely to improve cerebellar source localization, as discussed earlier, but will not be as crucial as in EEG. In terms of sensitivity, Samuelsson et al. (2020) also show that MEG is particularly sensitive to the posterior surface of the cerebellum. Paradigms that elicit activity close to the posterior surface of the cerebellum, such as the air-puff paradigm (e.g. Lin et al., 2019) and potentially touch/omission paradigms (e.g. Andersen and Lundqvist, 2019, may thus be especially worthwhile to investigate). Samuelsson et al. (2020) also show that EEG may be more sensitive to the anterior lobes of the cerebellum.

### 4.9 Summary and general recommendations

There are thus several strategies to employ to detect cerebellar activity. For any paradigm, however, one should increase the signal-to-noise ratio by acquiring as many trials as feasible. This necessitates a relatively simple paradigm without too many conditions. The results can be validated by ascertaining that any motor- or somatosensation-related responses arise from ipsilateral cerebellum. However, this requires both sides (e.g. left and right hands) to be tested – running counter to the idea of reducing the number of conditions. Experimental designs must therefore be optimized between these competing considerations.

### 4.10 MEG’s sensitivity to other structures outside neocortex

In this section, we briefly consider evidence for the sensitivity of MEG and EEG to structures outside of neocortex. Our intention with this is to dispel the notion that MEG and EEG are exclusively generated by pyramidal cells near the surface of the cerebral cortex.

The auditory brainstem response is perhaps the most well-known evidence that sensors on the scalp are capable of measuring subcortical activity (Jewett et al., 1970). The auditory brainstem responses consists of responses to brief auditory stimuli, generated sequentially by the cochlea, auditory nerve, superior olivary complex, lateral lemniscus, and inferior colliculus. It is routinely measured in the clinic with scalp electrodes, as a hearing test or measure of neural integrity. MEG sensors have also been able to capture the auditory brainstem response in experimental settings (Erné and Hoke, 1990; Parkkonen et al., 2009). It must be emphasized however that this requires many trials. Parkkonen et al. (2009) for instance used 16,000 trials, acquired over 30 ​min.

Ruzich et al. in their recent review (2019) found 37 MEG studies between the years 2005–2018 that revealed robust hippocampal activity. Similarly, Pizzo et al. (2019) found evidence that using independent component analysis (ICA) hippocampal and amygdala activations could be found with MEG (6 out of 14 patients). Data from some patients (4 out of 14) even revealed evidence of a thalamic signal. These activations were independently verified using simultaneous intracranial EEG recordings. Even though less than half the patients had detectable signal, this demonstrates the possibility to record from otherwise deep regions. In another recent study (Müller et al., 2019), alpha band functional connectivity between thalamus and visual cortex was found in congenitally blind subjects. Especially the thalamic findings are interesting, since it is still controversial whether thalamus is detectable by MEG at all, due to its location close to the centre of the brain and its morphology. However, supporting the validity of Pizzo et al.’s (2019) and Müller et al.’s (2019) findings, Attal and Schwartz (2013), using a combination of simulations and real data, showed that MEG is sensitive to signal arising from hippocampus, amygdala and thalamus. They emphasize the need to have anatomically precise source spaces, orientation-constrained dipoles (if the area has a preferred direction) and a realistic estimate of dipole moment densities in different regions. We echo them in our advice to use anatomically precise models of the cerebellum. Thus, there is nothing about the depth *per se* that leaves cerebellum outside MEG’s sensitivity range.

## 5 Conclusion

We conclude that it is indeed possible to detect EEG and MEG signals from the human cerebellum. Many studies using diverse methodologies have showcased EEG and MEG signals in different sensory domains such as audition, vision and somatosensation and during movements. There is also MEG-based evidence of the cerebellum being involved in more cognitive operations such as updating and maintaining sensory expectations, and in decision making.

Some limitations do exist at the moment however. The prime one is that the signal-to-noise ratio is low due to the larger distance between much of the cerebellum and the sensors (compared to the cerebrum). This means that we are likely to miss true activations of the cerebellum if the signal-to-noise ratio is low. Under favourable circumstances, e.g. high number of trials, optimized paradigms, facilitating coupling approaches, suppression of cortical activity, etc., this review indicates that cerebellar activation *can* be detected, just as many other deeper brain structures can, e.g. hippocampus, amygdala and thalamus. Even when we robustly detect cerebellar activation, however, we still face the limitation of spatial resolution - with EEG and MEG it is hard to detect where exactly within the cerebellum we are. More precise anatomical models of the cerebellum may be useful for constraining the source reconstructions possible with EEG and MEG.

EEG and MEG studies of the cerebellum however have the immense utility of being able to resolve brain activity as it unfolds in real time compared to the sluggish responses of fMRI. This may be paramount in understanding the complexities and details of cerebellar function and dysfunction.
